# The impact of the illness label ‘gout’ on illness and treatment perceptions in Māori (Indigenous New Zealanders)

**DOI:** 10.1186/s41927-020-00120-z

**Published:** 2020-04-15

**Authors:** Nicola Dalbeth, Meihana Douglas, Kate MacKrill, Leanne Te Karu, Maria Kleinstäuber, Keith J. Petrie

**Affiliations:** 1 Department of Medicine, Faculty of Medical and Health Sciences, University of Auckland, 85 Park Rd, Grafton, Auckland, New Zealand; 2 Department of Psychological Medicine, University of Auckland, Auckland, New Zealand; 3 Ngā Kaitiaki o te Puna Rongoā o Aotearoa, Taupō, New Zealand; 4 Department of Psychological Medicine, Dunedin School of Medicine, University of Otago, Dunedin, New Zealand

**Keywords:** Gout, Urate, Arthritis, Illness perceptions

## Abstract

**Background:**

Despite contemporary advances in understanding pathogenesis and effective management of gout, beliefs about the disease continue to be focused on gout as a self-inflicted illness. The illness label itself may contribute to inaccurate perceptions of the disease and its management. In Aotearoa/New Zealand, Māori (Indigenous New Zealanders) have high prevalence of severe gout. The aim of this study was to examine the impact of the illness label ‘gout’ on perceptions of the disease and its management for Māori.

**Methods:**

Māori supermarket shoppers (*n* = 172) in rural and urban locations were recruited into a study examining the perceptions about arthritis. Participants were randomised 1:1 to complete a questionnaire examining the perceptions of the same illness description labelled as either ‘gout’ or ‘urate crystal arthritis’. Differences between the two illness labels were tested using independent sample t-tests.

**Results:**

‘Gout’ was most likely to be viewed as caused by diet, whereas ‘urate crystal arthritis’ was most likely to be viewed as caused by aging. ‘Urate crystal arthritis’ was seen as having a wider range of factors responsible for the illness, including stress or worry, hereditary factors and chance. ‘Gout’ was less likely to be viewed as having a chronic timeline, and was perceived as being better understood. Dietary management strategies were seen as more helpful for management of the gout-labelled illness.

**Conclusions:**

This study has demonstrated that for Māori, Indigenous New Zealanders who are disproportionately affected by gout, the illness label influences perceptions about gout and beliefs about management.

## Background

Although presenting as an intermittently flaring condition, gout is a chronic disease caused by deposition of monosodium urate crystals [1]. Long-term urate-lowering therapy, available in the form of inexpensive, generic medications such as allopurinol, is effective in achieving dissolution of crystals, gout flare prevention and tophus regression [2–4]. However, many people with gout are not prescribed urate-lowering therapy, even when there are clear indications for this therapy [5]. Furthermore, urate-lowering therapy discontinuation and prolonged breaks in therapy are common [6].

Throughout Western history, gout has been depicted as a self-inflicted illness of lifestyle excess [7, 8]. Although dietary risk factors exist for development of disease, many other non-dietary factors (e.g. advancing age, male sex, kidney disease, medications, genetic variation) also contribute to the development of disease [9–11]. Despite contemporary advances in understanding pathogenesis and effective management of gout, prevailing community beliefs about this illness continue to be focused on diet as the dominant cause and strategy for disease management [12, 13]. These lay beliefs are frequently reflected in health care providers’ beliefs about gout and approaches to gout management [14]. Qualitative research has highlighted the negative impact of these illness perceptions on people with gout, which can contribute to embarrassment, stigma and avoidance of effective health behaviour or self-management strategies [15–17].

Illness labels are an important starting point for the formation of illness perceptions and can have an important impact on people’s beliefs about illness and decisions about management strategies [18]. Research shows patients construct organised cognitive representations of their illness that share a common structure and guide behaviour aimed at managing the illness [19, 20]. We have recently reported a study of supermarket shoppers in Aotearoa/New Zealand to examine the influence of the illness label ‘gout’ on the perceptions of the illness and its management [21]. This study indicated that compared with a pathophysiological illness label (‘urate crystal arthritis’), the gout-labelled illness was perceived as being more likely caused by the patient’s own behaviour through poor diet and overconsumption of alcohol. The gout-labelled illness was seen as less serious, less chronic, and more embarrassing. Management of the gout-labelled illness focused on dietary interventions, while the urate crystal arthritis-labelled illness was perceived as requiring long-term medication.

Māori (the Indigenous people of Aotearoa/New Zealand) have high prevalence of gout, with the disease affecting almost 10% of the adult population, and one third of Māori men over the age of 65 years [22, 23]. Furthermore, Māori with gout have earlier onset of disease compared to people of New Zealand European ethnicity, and have more severe disease with higher flare frequency and activity limitation [24]. In our prior supermarket study [21], the majority of participants were of New Zealand European ethnicity, and only 3.5% of participants were Māori. Therefore, it is unknown how the illness label impacts on Māori, who are disproportionally affected by gout. The aim of this study was to examine the impact of the illness label of ‘gout’ on perceptions of the illness and its management for Māori.

## Methods

The study methodology was identical to our prior study of supermarket shoppers which examined the effect of renaming gout on illness and treatment perceptions [21], with the exception of the research location and study participants. In this study, the research location was supermarkets in rural (Wairoa, New Zealand) and urban (Auckland, New Zealand) locations with large Māori communities.

Briefly, a stand was constructed outside a local supermarket and shoppers were asked whether they would like to participate in a study examining public perceptions of different types of arthritis, without specific mention of gout. Participants were provided with a written information sheet, and if they agreed to participate in the study, completed one of two versions of a written anonymous research questionnaire to complete. As this was an anonymous survey study, signed written consent could not be obtained. Rather, the participant information sheet stated that “By completing the anonymous survey, you are agreeing to participation in this study.” An assistant unrelated to the study had placed the ‘urate crystal arthritis’ or ‘gout’ research questionnaires in a random order from a computer-generated randomized list. Following completion of the questionnaire, participants received a NZ$10.00 supermarket-shopping voucher. The study complied with the Declaration of Helsinki. Ethics approval for the study was provided by the University of Auckland Human Participants Ethics Committee (Ref. 017777). Māori ethnicity was self-identified, consistent with New Zealand Ministry of Health standards for ethnicity data protocols [25].

Participants were provided with one of two versions of the questionnaire in English that either used the term ‘urate crystal arthritis’ or ‘gout’ as the label for the illness. A brief disease description was provided at the start of the questionnaire: *“Urate crystal arthritis /Gout is a type of arthritis that causes repeated attacks of excruciating joint pain. At the time of the attack, the joint is hot, red and tender. The affected person may be unable to sleep, walk, or go to work. Attacks of arthritis come on suddenly and can last for two weeks at a time. Occasionally, the joint can become damaged and deformed. Anti-inflammatory medications can be used to treat the attacks of arthritis. A long-term daily medication can prevent future attacks and joint damage. This questionnaire asks you about your beliefs about what it would be like to be diagnosed with urate crystal arthritis****/****gout. Even though you do not have this condition or may not know of anyone with this condition, we are interested in your perceptions about the condition.*”

Participants were then asked to complete a questionnaire examining their causal beliefs, illness perceptions, strategies for managing the illness, and demographic features (age, sex, ethnicity). Causal beliefs were examined by asking participants to rate, on a 5-point scale from ‘strongly disagree’ to ‘strongly agree’, whether the following factors were important in causing the illness: stress; hereditary; diet; aging; alcohol use; environmental pollution; chance or bad luck; a germ or virus; the persons own behaviour. Items from the Brief Illness Perception Questionnaire were used to assess beliefs about the illness [26]. The questionnaire assesses consequences; timeline beliefs; personal control; medication control; likelihood of experiencing symptoms; concern about illness; seriousness of condition; understanding of the illness; embarrassment about being diagnosed with the illness; and how much the illness would affect you emotionally. Items are rated on an 11-point scale (from 0 to 10) with relevant anchors for each. The scale has been widely used and shown to be a valid and reliable measure [27]. Strategies for managing the illness were examined by rating, on a 11 point scale from 0 ‘Wouldn’t help at all’ to 10 ‘Very likely to help’, the value of the following strategies for controlling the condition: changing to a healthier diet; using long-term medications; managing stress; getting regular exercise; stopping or restricting alcohol; using alternative medicine; losing weight.

The study plan pre-specified analysis of Māori participants. Of the 473 participants providing completed questionnaires, 172 (37%) Māori participants were recruited, and these participants were included in the analysis of Māori participants. The sample size of 170 participants in total was based on our prior study of illness labels of ‘urate crystal arthritis’ and ‘gout’ [21]; this sample size allowed detection of an at least 2 point difference in the Brief Illness Perception Questionnaire items between the gout and urate crystal arthritis groups with power of 0.9 and alpha of 0.05. Data were analyzed using the Statistical Package for Social Sciences (SPSS) v25. Differences between ‘urate crystal arthritis’ and ‘gout’ groups on causal beliefs, illness perceptions and strategies for managing the condition were tested using independent sample t-tests. This analysis method for independent groups was used on the basis that each of the Likert scales were ordinal with conceptually equal spacing between categories in the same domain and that the sample size was sufficiently large (> 30) so as to be robust to departures from normality.

## Results

### Participant characteristics

Of the 172 Māori participants; 89 (51.7%) responded for the gout-labelled illness and 83 (48.3%) for the urate crystal arthritis-labelled illness. The mean (standard deviation [SD]) age of the Māori participants was 42 (13) years. There were 125 (72.7%) women, 81 (47.1%) living in a rural setting, and 91 (52.9%) living in an urban setting. Randomization groups were well matched for age, sex, and rural/urban residence (Table 1).

**Table 1:** Participant characteristics

	Gout-labelled illness, *n* = 89	Urate crystal arthritis-labelled illness, *n* = 83	All participants, *n* = 172
Age, mean (SD) years	42 (15)	41 (17)	42 (15)
Female sex, n (%)	65 (73%)	60 (72%)	125 (73%)
Rural setting, n (%)	41 (46%)	40 (48%)	81 (47%)

### Causal beliefs

Causal beliefs for each illness label are shown in Table 2. The gout-labelled illness was significantly more likely to be viewed as caused by diet compared to the urate crystal arthritis-labelled illness which was most likely to be viewed as caused by aging. Urate crystal arthritis was seen as having a wider range of factors as responsible for the illness, with aging, stress or worry, hereditary factors, chance and pollution more likely to be viewed as causes of ‘urate crystal arthritis’ compared with ‘gout’.

**Table 2:** Likely causal factors for ‘gout’ and ‘urate crystal arthritis’. Data are shown as mean (SD)

	Gout-labelled illness	Urate crystal arthritis-labelled illness	P
Diet	4.2 (1.0)	3.7 (1.0)	0.003
Alcohol	3.8 (1.3)	3.5 (1.2)	0.16
Own behaviour	3.2 (1.3)	3.2 (1.3)	0.91
Aging	3.5 (1.2)	4.1 (0.9)	0.001
Stress or worry	3.2 (1.2)	3.8 (1.1)	0.003
Heredity	3.4 (1.4)	3.9 (0.9)	0.005
Chance	2.2 (1.3)	3.1 (1.3)	0.001
Pollution	2.8 (1.2)	3.3 (1.2)	0.01
Germ or virus	2.6 (1.2)	2.9 (1.1)	0.087

### Illness perceptions

Illness perceptions for ‘gout’ and ‘urate crystal arthritis’ are shown in Table 3. ‘Gout’ was less likely to be viewed as having a chronic timeline than ‘urate crystal arthritis’ (mean (SD) for ‘gout’ 6.9 (2.8) and for ‘urate crystal arthritis’ 7.9 (2.4), *P* = 0.013). Respondents felt they understood ‘gout’ better than ‘urate crystal arthritis’ (mean (SD) for ‘gout’ 6.3 (3.1) and for ‘urate crystal arthritis’4.4 (3.3), *P* = 0.001). The other illness perceptions – the consequences of the illness on the patient’s life, seriousness of the condition, personal control, the experience of symptoms, illness concern, embarrassment, or how much the illness would affect the patient emotionally – did not differ significantly between the two groups (Table 3).

**Table 3:** Illness perceptions for ‘gout’ and ‘urate crystal arthritis’. Data are shown as mean (SD)

	Gout-labelled illness	Urate crystal arthritis-labelled illness	P
Consequences (10 = severely affects)	7.4 (2.6)	7.7 (2.7)	0.46
Timeline (10 = forever)	6.9 (2.8)	7.9 (2.4)	0.013
Personal control (10 = extreme amount)	6.0 (3.1)	5.4 (2.8)	0.19
Treatment control (10 = extremely helpful)	7.4 (2.3)	6.7 (2.8)	0.08
Identity (10 = many severe symptoms)	5.8 (3.1)	5.8 (2.6)	0.95
Concern (10 = extremely concerned)	7.6 (2.7)	7.5 (2.9)	0.93
Understanding (10 = very clearly)	6.3 (3.1)	4.4 (3.3)	< 0.001
Embarrassment (10 = very embarrassed)	5.6 (3.7)	5.1 (3.8)	0.42
Emotions (10 = extremely affected)	7.3 (2.7)	7.4 (2.6)	0.71

### Strategies for managing the illness

Beliefs about strategies for managing ‘gout’ and ‘urate crystal arthritis’ are shown in Table 4. Changing to a healthier diet was perceived as more helpful for ‘gout’ compared to ‘urate crystal arthritis’ (mean (SD) for ‘gout’ 8.5 (2.3) and for ‘urate crystal arthritis’ 7.3 (2.7), *P* = 0.003). Participants also viewed stopping or restricting alcohol use as more helpful for ‘gout’ than ‘urate crystal arthritis’ (mean (SD) for ‘gout’ 8.1 (2.8) and for ‘urate crystal arthritis’ 7.0 (3.1), *P* = 0.017). For ‘gout’, changing to a healthy diet and stopping or restricting alcohol were scored as the most important strategies. For ‘urate crystal arthritis’, changing to a healthy diet and long-term medication were scored as the most important strategies. There were no differences between ‘gout’ and ‘urate crystal arthritis’ in perceptions that adopting regular exercise, losing weight or taking long-term medications would be helpful for managing the illness.

**Table 4:** Beliefs about strategies for managing for ‘gout’ and ‘urate crystal arthritis’. Data are shown as mean (SD)

	Gout-labelled illness	Urate crystal arthritis-labelled illness	P
Need for long-term medication	6.7 (3.3)	7.2 (2.7)	0.24
Managing stress	6.9 (2.9)	7.0 (2.9)	0.90
Regular exercise	7.4 (3.0)	7.1 (2.8)	0.47
Stopping or restricting alcohol	8.1 (2.8)	7.0 (3.1)	0.017
Changing to a healthy diet	8.5 (2.6)	7.3 (2.8)	0.003
Using alternative medicine	7.3 (2.9)	7.1 (2.5)	0.76
Losing weight	7.2 (3.4)	6.8 (3.2)	0.42

## Discussion

This study has demonstrated that for Māori, Indigenous New Zealanders who are disproportionately affected by gout, the label impacts on illness perceptions and some beliefs about management. The causal beliefs and management strategies for the gout-labelled illness are consistent with widely-held beliefs that gout is an illness primarily caused by self-inflicted dietary excess, whereas the urate crystal arthritis-labelled illness promotes a broader view of the causal factors and disease management.

The findings of this study are similar to our prior study in an urban setting, which included few Māori participants. In line with our previous study of supermarket shoppers [21], and prior studies of New Zealanders with gout [16, 28], the most common causes of the gout-labelled illness were perceived to be diet and alcohol intake. In contrast, the most common causes of the urate crystal arthritis-labelled illness were perceived to be biological variables, such as aging and heredity factors. Consistent with beliefs of illness causation, both supermarket studies have shown that a pathophysiological illness label leads to broader beliefs about illness management strategies and increases the view of the condition as a chronic condition. Some differences were also observed in the two studies. The initial study with predominantly New Zealand European participants demonstrated that the gout-labelled illness was viewed as a less serious and more embarrassing condition than the urate crystal arthritis-labelled illness [21]. This was not the case for this study of Māori participants, who viewed both ‘gout’ and ‘urate crystal arthritis’ as a serious condition. These differences may reflect the higher prevalence and major impact of the disease in Māori, due to the severe pain and disability caused by gout [16].

This study also showed large differences in perceptions of illness understanding between the labels, with understanding about ‘gout’ significantly higher than understanding about ‘urate crystal arthritis’. Reducing familiarity with an illness label may provide health professionals with the opportunity to introduce new concepts about illness causation and management strategies to patients. There is also a risk that a more complex label might also increase uncertainty about the disease and its treatment.

Our findings align with prior qualitative research studies of Māori participants with gout. In a study guided by kaupapa Māori principles (defined as research by Māori, for Māori and with Māori [29]), gout had a huge, negative impact on the lives of participants [16]. All participants believed or had been informed that gout is caused by food and/or drink, which led to feelings of self-blame and blame from partners and employers. In a focus group study of Māori, understanding about gout was obtained from many sources, including health care professionals, family and friends, and personal experiences [30]. The research highlights the need for further work on perceptions of gout in Māori patients using both established questionnaires and the use of alternative strategies for assessing illness perceptions, such as drawings [31].

Previous clinical trials in other diseases have demonstrated that changing illness perceptions can have positive effects on health outcomes. For example, in young adults with asthma, a targeted text message programme that changed illness perceptions led to improved medication adherence [32]. Furthermore, a brief in-hospital illness perception intervention following myocardial infarction leads to earlier return to work and lower reports of angina symptoms post- discharge [33]. Future studies will examine the effects of an alternative illness label on the perceptions and outcomes in people with gout.

We acknowledge the study limitations. A potential limitation is the use of a community sample, rather than a group of people with a diagnosis of gout. Specifically, those experiencing a gout flare were unlikely to be recruited into the study, as the severe pain of a gout flare often limits ambulation which would be required for supermarket shopping. The study population of supermarket shoppers was also relatively young and predominately female, and the views of older men may not be fully represented. Although the demographics of the study population may not reflect those of people with gout, we consider the recruitment strategy appropriate, as influencing community attitudes about the illness is likely to have substantial benefits for affected individuals, who frequently experience blame and negative comments from partners and employers at the time of a gout flare [15, 16]. Furthermore, Māori with gout identify whānau (family) as an important source of health information [16]. Work is also needed to examine the impact of such a name change on health care providers’ beliefs and behaviours. Myths about gout can be perpetuated in health interactions [34], and changing the illness label may challenge health care providers about their own beliefs and provide a framework to initiate different conversations with patients and whānau about the causes of and management strategies for the illness. A strength of this study was the recruitment of a large group of Indigenous people. However, it is important to recognise that the study findings may not be generalizable to Indigenous people in countries other than Aotearoa/New Zealand, or in people who speak languages other than English.

## Conclusions

For Māori, Indigenous New Zealanders who are disproportionately affected by gout, causal beliefs and management strategies for a gout-labelled illness are consistent with a disease that is primarily caused by self-inflicted dietary excess. A pathophysiological illness label may promote more complex causal beliefs including a longer timeline for the illness, and reduce perceptions that dietary modification and alcohol restriction are the main strategies for management.

## Data Availability

The datasets used and/or analysed during the current study are available from the corresponding author on reasonable request.

## Abbreviations

SD: Standard deviation
SPSS: The Statistical Package for Social Sciences
